# Experimental Sepsis Impairs Humoral Memory in Mice

**DOI:** 10.1371/journal.pone.0081752

**Published:** 2013-11-28

**Authors:** Christian Pötschke, Wolfram Kessler, Stefan Maier, Claus-Dieter Heidecke, Barbara M. Bröker

**Affiliations:** 1 Institute of Immunology and Transfusion Medicine, University Medicine Greifswald, Greifswald, Germany; 2 Department of Surgery, University Medicine Greifswald, Greifswald, Germany; University of São Paulo, Brazil

## Abstract

Patients with sepsis are often immune suppressed, and experimental mouse models of sepsis also display this feature. However, acute sepsis in mice is also characterized by a generalized B cell activation and plasma cell differentiation, resulting in a marked increase in serum antibody concentration. Its effects on humoral memory are not clearly defined. We measured the effects of experimental sepsis on long-term immunological memory for a defined antigen: we induced colon ascendens stent peritonitis (CASP) 8 weeks after 2 rounds of immunization with ovalbumin. Four weeks later, the antigen-specific bone marrow plasma cell count had doubled in immunized non-septic animals, but remained unchanged in immunized septic animals. Sepsis also caused a decrease in antigen-specific serum antibody concentration. We conclude that sepsis weakens humoral memory by impeding the antigen-specific plasma cell pool’s development, which is not complete 8 weeks after secondary immunization.

## Introduction

Sepsis is still associated with astoundingly high morbidity and mortality despite improvements in intensive care [1,2]. In sepsis, a hyper-inflammatory phase is followed by compensatory anti-inflammatory response syndrome (CARS), with the risk of lethal secondary infections [3,4]. Mortality from sepsis occurs mostly during this phase [5,6]. Besides its high acute mortality, sepsis has a poor long-term prognosis [7,8]. For example, post septic patients are more susceptible to cancer, heart disease and pneumonia [9]. The reasons are not known, but it is conceivable that sepsis damages humoral memory, thereby facilitating infections with pathogens against which a protective immunity had already been built.

Humoral memory consists of resting memory B cells that are rapidly activated after repeated contact with antigen, and long-lived plasma cells that reside in survival niches of the bone marrow [10,11] and secrete protective antibodies over a long period of time [12]. In a classical immune response, plasma cells and memory B cells are generated in the germinal center reaction [13-15]. Approximately 10-20% of plasma cells formed during the germinal center reaction become long-lived plasma cells also known as memory plasma cells [16-18]. The number of long-lived plasma cells is restricted by a limited number of plasma cell niches in bone marrow [19,20]. Upon each immune response, newly formed plasma cells compete for the few survival niches [21]. In a further restriction, long-lived plasma cells undergo apoptosis upon cross-linking of their FcγRIIB-receptors with, for example, immune complexes [22,23].

Sepsis suppresses the adaptive immune system. This has been demonstrated for the priming of B cell- and T cell responses and for T cell effector functions [24-26]. Whether sepsis also impinges on established humoral memory is not known, but it is well conceivable. Existing models of immunological memory predict different effects of sepsis. In septic mice, B cells are strongly activated, resulting in large numbers of plasma cells which ensure an increase in serum immunoglobulin concentrations [26]. In this way, sepsis could ‘overwrite’ humoral memory by outcompeting previously established plasma cells. On the other hand, sepsis could directly affect the survival niches of long-lived bone marrow plasma cells. Eosinophil granulocytes and megakaryocytes are important components of the long-lived bone marrow plasma cell survival niches [27,28]. The enhanced disseminated intravascular coagulation that occurs in sepsis mobilizes megakaryocytes [29-31], and the microbial products activate eosinophils [32-34]. If sepsis changes the composition of supporting cell types in the survival niche, long-lived plasma cell populations could be affected and humoral memory could thereby be weakened. This is how sepsis would dampen the protection provided by vaccination, or in general, the adaptive protection against pathogens [35-37], resulting in susceptibility to further infections. This could contribute to the increased on-going mortality risk after sepsis, detected epidemiologically in humans up to 5 years later [7,8]. However, alternative models imply that sepsis strengthens the pre-existing immunological memory. According to this notion, memory B cells maintain protective serum antibody concentrations because they are activated via their TLRs by the numerous microbial components that flood the system during sepsis [38,39]. To test these hypotheses, we have established immune memory in mice by vaccinating and boosting with a defined antigen. Following this, poly-microbial peritonitis was induced as a model of sepsis. We found that sepsis reduced antigen-specific serum IgG as well as the number of antigen-specific antibody secreting cells in the bone marrow.

## Materials and Methods

### Animal experiments and ethics statement 

Female C57BL/6 wild type mice (Charles River, Sulzfeld, Germany) were housed in a conventional, temperature-controlled animal facility with a 12-hour light/12-hour dark cycle and provided with food and water ad libitum. All experiments were performed according to the German animal safety regulations and approved by the animal ethics committee of the local animal protection authority (Regional Authority for Agriculture, Food Safety and Fishery of Mecklenburg-Vorpommern). All efforts were made to minimize suffering. Mice were immunized i.p. at 6 weeks (wk) with 100 µg trinitrophenlyl-13-ovalbumin (TNP-13-OVA, Biosearch Technologies, Inc, CA) and 50 µg ovalbumin (OVA) in alum (Pierce, Rockford, IL) and boosted 3 wk later. Eight weeks later, CASP surgery was performed as described previously [40,41]. Briefly, mice were anaesthetized with ketamine/xylazine (100 mg/10 mg per kg bodyweight) and an 18 G stent was implanted into their colon ascendens. Animals received Buprenorphin s.c. for pain control. Two or 4 week after CASP, surviving mice were deeply anaesthetized and blood was recovered. Animals were then sacrificed, their spleens were explanted and meshed through a 70 µm nylon-strainer. All femurs and tibiae were cut open at the very distal ends with a spatula, and cells were eluted with 10 ml PBS each using a syringe. The cell suspensions were depleted of erythrocytes using a brief incubation with distilled water, immediately washed twice in PBS, and re-suspended in cell culture medium (RPMI 1640, containing 10% FCS, glutamine, penicillin, and streptomycin). 

### Antibody screening assays

Total serum IgM and IgG concentrations were determined using the Milliplex Mouse Immunoglobulin Isotyping Immunoassay (Millipore, Billerica, MA) according to the manufacturer’s instructions. The samples were measured with the Luminex 200 System (Bio-Rad Laboratories, Munich, Germany).

OVA-specific antibodies in serum were determined by coating MaxiSorb-microtiterplates (Nunc, Roskilde, Denmark) with OVA (10 µg/ml) and blocking with 10% FCS/PBS. Diluted serum samples were incubated and bound antibodies were detected with peroxidase-conjugated goat-anti-mouse-IgM (Dianova, Hamburg, Germany, 1:30000 in 10% FCS/PBS) or -IgG (SouthernBiotech, Birmingham, AL, 1:30000 in 10% FCS/PBS) and OptEIA™ TMB Substrate Reagent (BD Biosciences, San Jose, CA). Optical density was measured at 450 nm. In the ELISA, anti-OVA and anti-TNP-BSA antibodies were measured against a standard serum.

### ELISPOT

MultiScreenHTS-IP Filterplates (Millipore, Billerica, MA) were either coated with 100 µl OVA (50 µg/ml) or 100 µl TNP-13-BSA (50 µg/ml). Free protein binding sites were blocked with 2% FCS/PBS. In all, 1×10^6^ splenocytes or bone marrow cells were seeded into the cavities in serial dilution. After thorough washing, bound antibodies were detected with biotin labeled rat-anti-mouse-IgM (BD, Heidelberg, Germany 1 µg/ml) or goat-anti-mouse-IgG (Dianova, Hamburg, Germany 1 µg/ml), peroxidase-labeled Streptavidin (Dianova, Hamburg, Germany 5 µg/ml) and AEC-Substrate (Sigma-Aldrich, Steinheim). Spots were enumerated with the Immunospot Series 5 Versa ELISPOT Analyzer (CTL Europe GmbH, Bonn, Germany) and the ImmunoCapture^TM^ Image Acquisition software. Data were analyzed with ImmunoSpot^®^ Analysis software V5. 

### Statistical analysis

Statistical analyses were performed using GraphPad Prism 5 for Windows (GraphPad software, San Diego, CA). We used one-way ANOVA with the Bonferroni’s posttest for selected pairs. P-values < 0.05 were considered to be significant. Analyses with a non-parametric two-tailed Man-Whitney U test gave similar results (table S1).

## Results and Discussion

### Experimental mouse model of humoral memory

**Figure 1:**
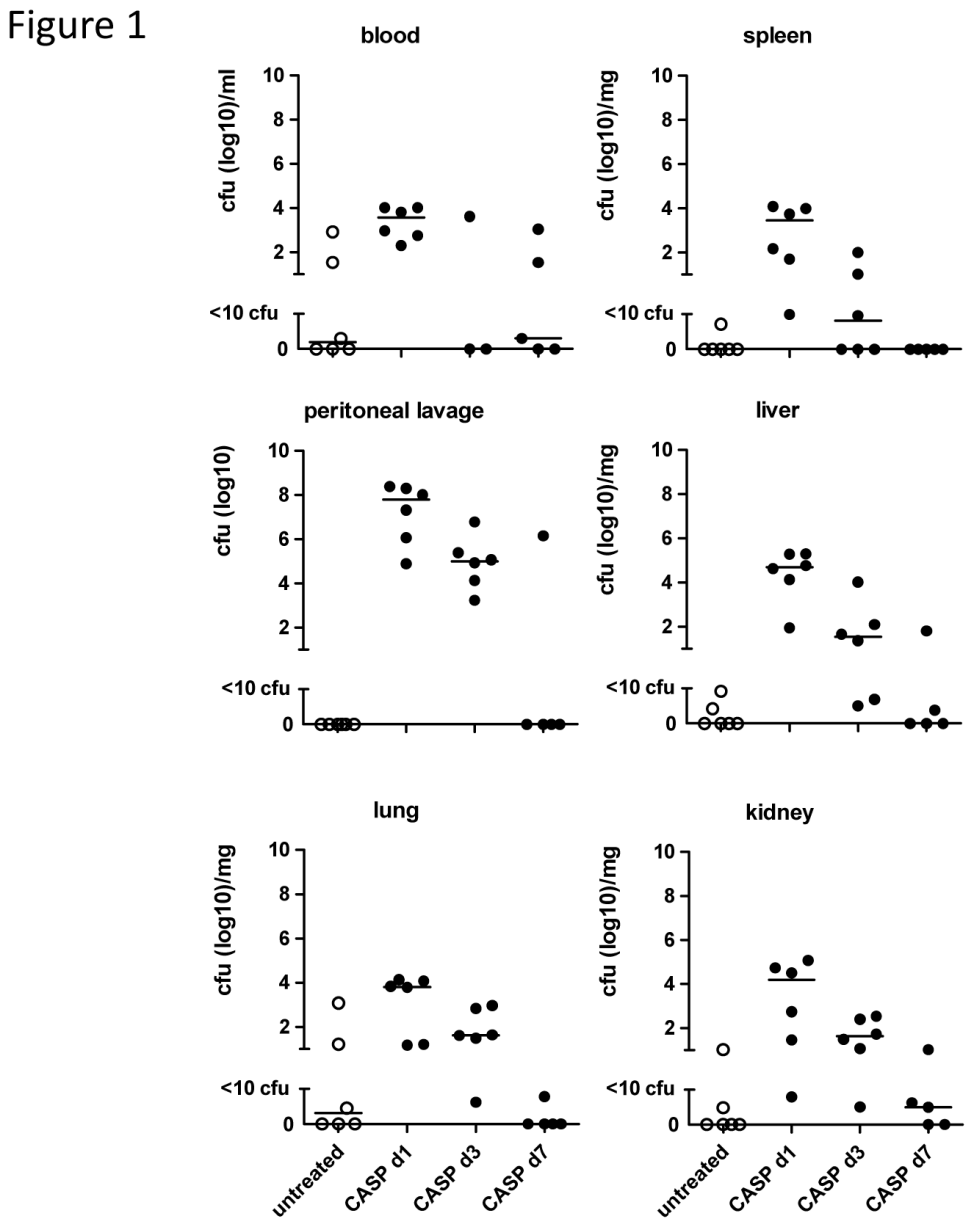
Bacterial dissemination and clearance during experimental sepsis. C57BL/6 mice were subjected to CASP (18G). At the indicated time points blood, spleen, peritoneal lavage, liver, lung, and kidney were harvested. Solid organs were homogenized with an ultrathorax. Liquids and organ suspensions were plated on agar-plates and incubated for 22 h, when cfus were enumerated. n=5-6 mice/group.

We established an experimental mouse model of humoral memory against which to measure the effects of sepsis. We immunized C57Bl/6 mice with OVA at 6 wk and performed a secondary OVA immunization at 9 wk. Ten and 12 week after the secondary immunization, we measured parameters of humoral memory. Against this background, we induced experimental sepsis by CASP 8 wk after the secondary immunization to assess humoral responses to sepsis. Thus the readout of memory took place two and 4 wk after sepsis induction. The CASP model has been developed to closely mimic the clinical situation of diffuse fecal peritonitis, a complication of abdominal surgery caused by intestinal leakage [41]. CLP, in contrast, resembles intra-abdominal abscess formation. The CASP model is clinically highly relevant, since sepsis and multi-organ failure are common in patients suffering from diffuse peritonitis [40]. The survival rate of CASP was 38% in the two long-term experiments described here. Different from CLP, there is no late mortality in CASP but animals who have survived the first 72 h usually remain symptom-free [40]. In CASP, endogenous bacteria rapidly disseminated systemically, but were cleared from the system around 7 d later (Figure 1), whereas in CLP bacterial release from the peritoneal focus is moderate and continues over an extended period of time [40]. Hence, early mortality in CASP is caused by hyper-inflammation.

**Figure 2:**
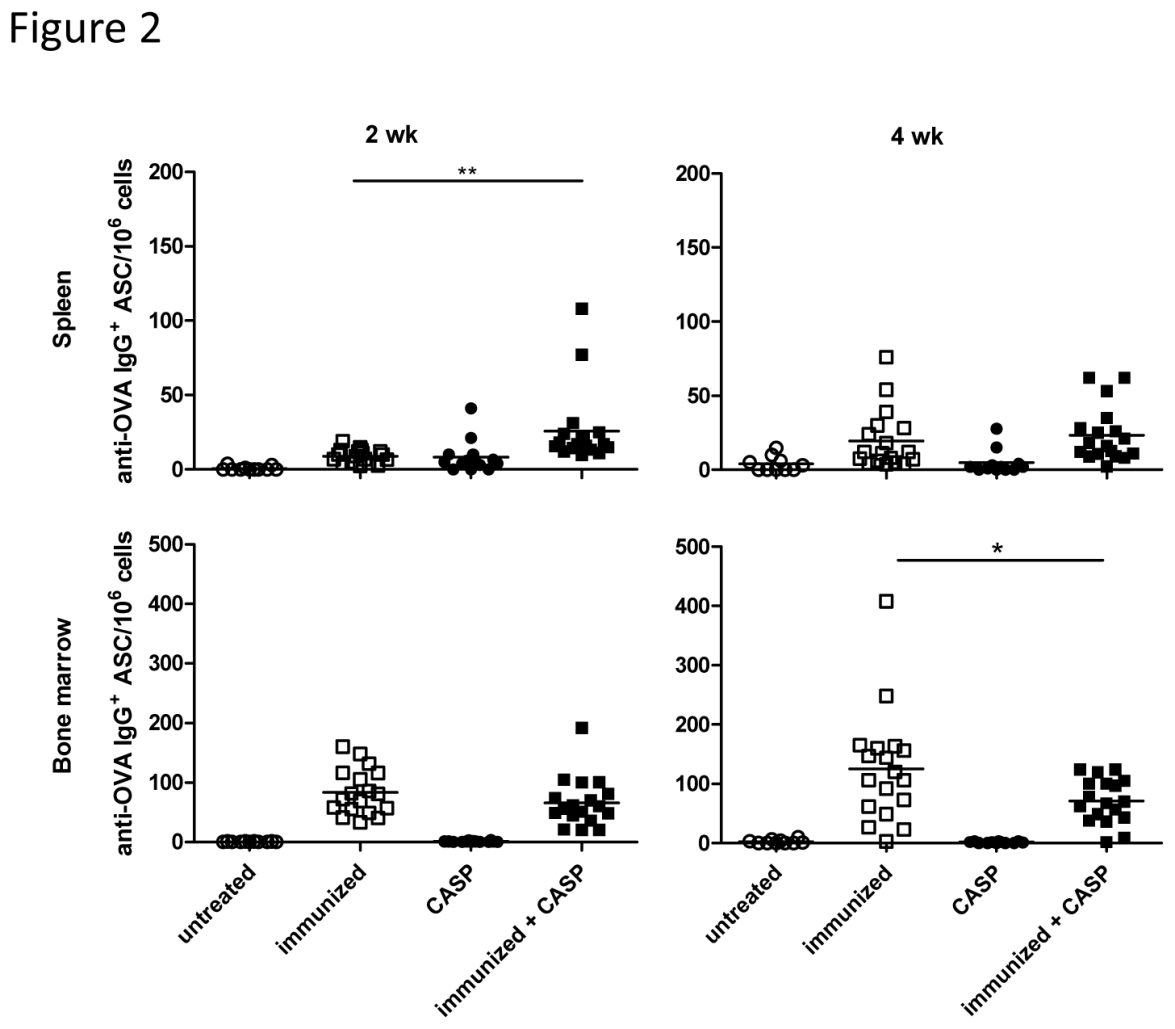
Experimental sepsis in OVA-immunized mice reduces OVA-specific IgG-ASCs. OVA-specific IgG-ASCs in spleen and bone marrow 2 and 4 wk after CASP (colon ascendens stent peritonitis) are shown here. C57BL/6 mice were immunized and boosted with OVA at 6 and 9 wk, respectively, and CASP was induced 8 wk after the secondary immunization. Control animals were either untreated, only immunized or only CASP induced. A combination of two independent experiments with the same trend is shown here. A statistical analysis was performed with the one-way ANOVA and Bonferroni’s post test for selected pairs. N = 10-18/group. *: p<0.05; **: p<0.01.

In our model of humoral memory OVA-specific bone marrow antibody-secreting cells (ASC) were abundant in the bone marrow at 10 wk after the secondary immunization and they further increased during the next two weeks (from 83.4±38.3 × 10^6^ to 125±94.2 × 10^6^; p = 0.09) This shows that the antigen-specific, long-lived bone marrow plasma cell population is not fully established by 10 wk after a secondary immunization (Figure 2, table S1). This could be due to a slow division of antigen-specific plasma cells in bone marrow. However, according to our current understanding, plasma cells are terminally differentiated and no longer divide [42]. This has already been called into question by Ahuja et al., who suspected a very limited capacity of plasma cells for cell division [43]. Alternatively, the migration of plasma cells from the periphery into the bone marrow after the secondary immunization might not be completed, despite the lengthy period following the boost immunization. Manz et al. showed that plasma cells appear in the bone marrow 10 days after the secondary immunization, thereafter remaining at a constant level for at least 3 months [44]. However in a further experiment, the same group also showed that the migration of the plasma cells into the bone marrow is completed only 2 months after a secondary immunization. Until this time point, some plasma cells still migrate into the bone marrow [10]. Thus, either a slow plasma cell division or a residual migration from the periphery, or both, might contribute to the increase in OVA-specific bone marrow ASCs between the two time points measured.

### Effects of experimental sepsis on humoral memory

**Figure 3:**
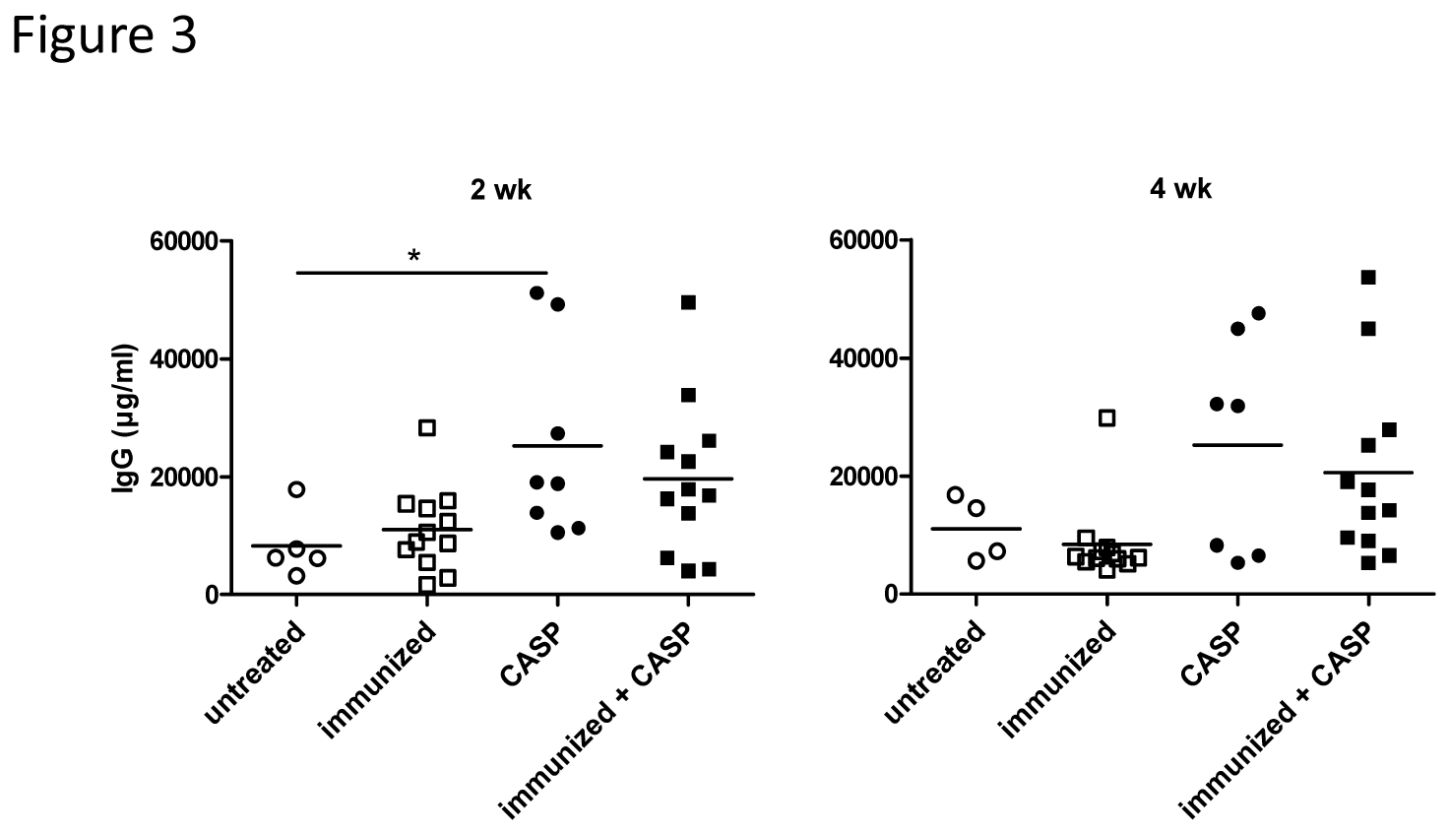
Total serum IgG concentration tends to increase after experimental sepsis. Serum IgM and IgG concentrations 2 or 4 wk after CASP (colon ascendens stent peritonitis) are shown here. C57BL/6 mice were immunized and boosted with OVA at 6 and 9 wk, respectively, and CASP was induced 8 wk after the secondary immunization. Control animals were either untreated, only immunized or only CASP induced. A statistical analysis was performed with the one-way ANOVA and Bonferroni’s post test for selected pairs. n=10-18/group. A single experiment is shown here.

Using our model of humoral memory, we induced experimental sepsis by CASP 8 wk after the secondary immunization. Two and 4 wk later, the total serum IgG concentration tended to be higher in septic animals than in the control animals (Figure 3), in agreement with previous observations of increased serum immunoglobulin concentrations after sepsis [26]. CASP leads to a clear transient increase of OVA-specific IgG^+^-ASC in the spleen (Figure 2, table S1). There are several possible explanations for this. In mice naïve and memory B cells express TLRs, hence the systemic dissemination of bacteria and bacterial products in sepsis can lead to their antigen-independent polyclonal activation [45-48]. First, memory B cells, located primarily in the splenic marginal zone, may be re-activated [38,39]. They could home to the bone marrow and replace some of the resident plasma cells. Second, naïve B1 cells are strongly activated during sepsis. These cells are usually poly-reactive and preferably bind to antigens with repetitive epitopes [49]. A minority of these B1 cells would be expected to cross-react with OVA. Interestingly, these cells do not establish themselves in the bone marrow (unpublished observation).

**Figure 4:**
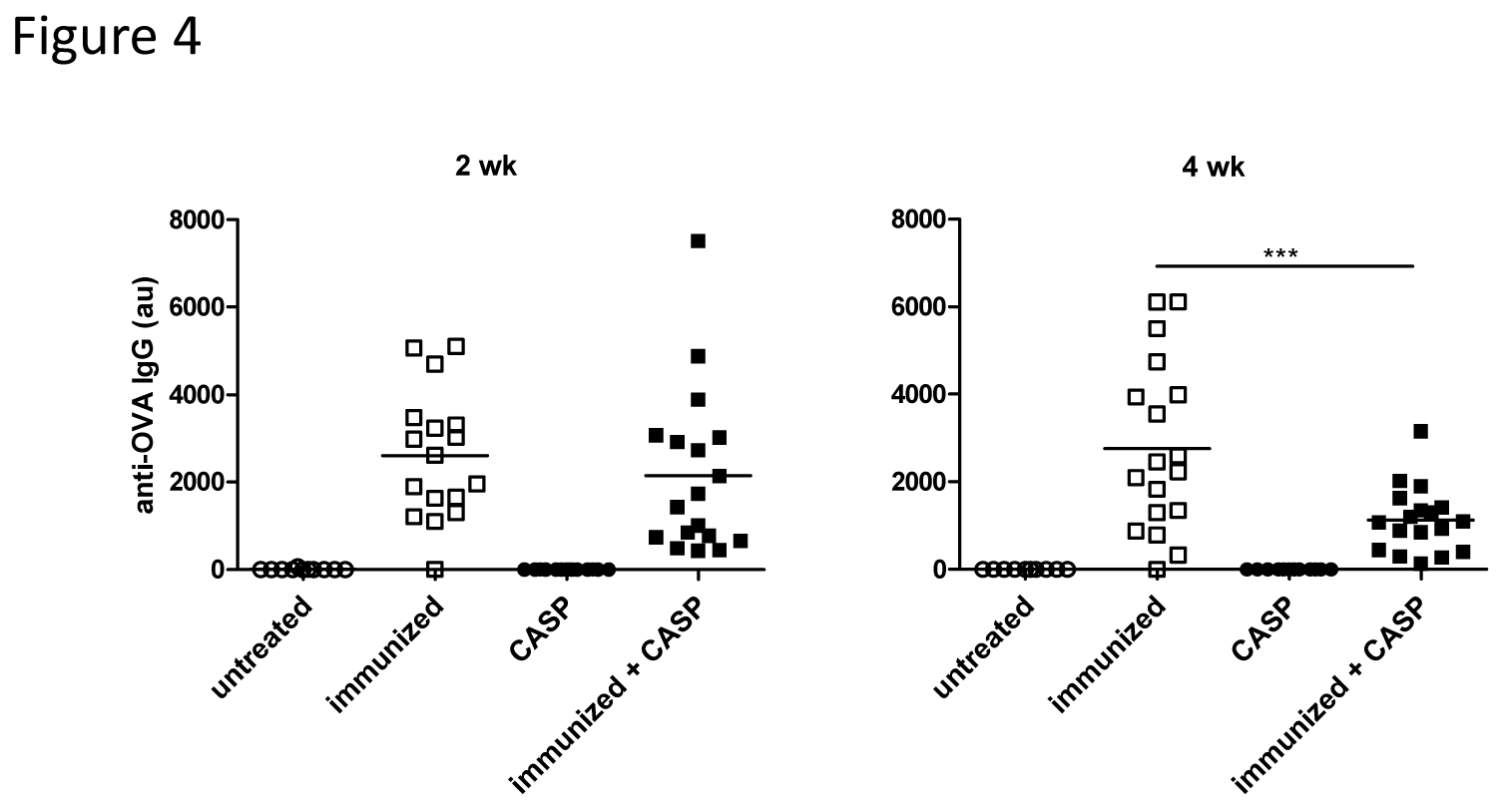
Experimental sepsis in OVA-immunized mice reduces OVA-specific serum IgG. OVA-specific serum-IgM and -IgG at 2 and 4 wk after CASP (colon ascendens stent peritonitis) are shown here (au = arbitrary units). C57BL/6 mice were immunized and boosted with OVA at 6 and 9 wk, respectively, and CASP was induced 8 wk after the secondary immunization. Control animals were either untreated, only immunized or only CASP induced. A combination of two independent experiments with the same trend is shown here. A statistical analysis was performed with the one-way ANOVA and Bonferroni’s post test for selected pairs. n=10-18/group. *: p<0.05; **: p<0.01.

In contrast to the spleen, there was no increase of OVA-specific ASCs in the bone marrow of immunized septic animals, where the majority of the ASCs was located. In fact, the number of bone marrow antigen-specific IgG-ASCs was significantly lower than in non-septic animals at 4 wk after sepsis (Figure 2, table S1), and in keeping with this, the serum OVA-specific antibody concentration was also much lower (Figure 4, table S1). TNP-specific ASCs and serum antibodies behaved in a similar manner (data not shown). The antigen-specific ASC count after sepsis had remained virtually unchanged between 10 and 12 wk after the secondary immunization, whereas the numbers in the non-septic animals had almost doubled. This indicates that sepsis can weaken the immunological memory. Using a similar model Xiang et al. observed an absolute decline of antigen-specific ASCs after “nonspecific” immunization with a complex antigen mixture. Their data also reveal an apparent late increase of antigen-specific plasma cells in animals not exposed to “nonspecific” immunization [23]. 

It is not known whether sepsis influences established humoral memory also in humans. However, since major surgery is also associated with suppression of the adaptive immune system [4], we have previously studied patients undergoing cardiopulmonary bypass surgery [50]. We found that serum IgG binding to typical recall antigens (tetanus- and diphtheria toxin) was not diminished but, on the contrary, modestly enhanced in accordance with the model of polyclonal activation of memory B cells by TLR ligands [38,39,50]. Similarly, Amanna and co-workers recently reported that neither inoculation of vaccinia virus nor multiple vaccination challenges over a time period of several months impaired established humoral memory [51]. Whether in humans humoral immunity is robust to severe sepsis as well, cannot be answered at present. The results of our animal experiments suggest that overwhelming systemic bacterial infection may be special regarding the impact on humoral memory.

The mechanism by which sepsis impedes the complete establishment of antigen-specific ASCs in the bone marrow at 12 wk after secondary immunization is unclear. Based on the current literature, sepsis would be expected to exert a number of stimulating effects on plasma cell survival. For example, eosinophil granulocytes are an important component of the survival niches [28], and when activated, support plasma cell survival particularly well in the bone marrow [52]. Because eosinophils are activated by TLRs to defend against bacteria [32-34], the large amounts of PAMPs in sepsis would be expected to activate eosinophils and thereby enhance the survival niche’s capacity for plasma cells. However, in our work, septic animals did not display the increase of long-lived plasma cells seen in non-septic animals and as such, eosinophil activation by sepsis would not explain this. Another important cell type in the bone marrow survival niche which could be affected by sepsis is the megakaryocyte, the precursor and producer of thrombocytes [27]. Both thrombopoiesis and megakaryopoiesis are tightly regulated by thrombopoietin (TPO).The administration of TPO leads to the accumulation and persistence of plasma cells in bone marrow [27] and TPO receptor (c-mpl)-deficient mice with impaired megakaryopoiesis have a reduced number of plasma cells in bone marrow. In sepsis, thrombocyte numbers decrease initially [53,54] due to disseminated intravascular coagulation [55], but the large amounts of IL-6 (amongst other cytokines) secreted during sepsis should increase TPO, and therefore accelerate thrombopoiesis [56,57], in the same way that LPS increases the TPO concentration via IL-6 during an acute phase response [58,59]. Presumably, the expected increase of thrombopoietin after sepsis should contribute to the ability of survival niches to take up more plasma cells in sepsis. In spite of this, the number of OVA-specific plasma cells in bone marrow did not increase in sepsis.

A failure to increase OVA-specific plasma cells after sepsis could be the result of an impaired migration from the periphery into the bone marrow. However, the possibility cannot be excluded that long-lived plasma cells die and are replaced with migrating plasma cells. If this occurs in balanced proportions, a change in cell numbers cannot be measured.

Another explanation for the lack of plasma cell pool expansion in our model might be a competition for the bone marrow niches between OVA-specific plasma cells and plasma cells with other specificities that originate during sepsis. On one hand, Bortnick et al. showed in a mouse model, that T cell independent B cell reactions can produce long-lived plasma cells, thereby also inducing an immunological memory [60]. On the other hand it is also possible, that T cell dependent B cell reactions in sepsis generate long-lived plasma cells that migrate into the bone marrow. Finally, it has been described that in animals deficient in FoxP3+ regulatory T cells (scurfy mice) plasma cells accumulate in the spleen, whereas they are diminished in the bone marrow [61]. Hence, Foxp3+ regulatory T cells influence the balance between plasma cell niches in the bone marrow and in the spleen. We have previously shown that in the early phase of sepsis Tregs lose their suppressive influence [62], their function being overridden by the overwhelming inflammation, which is in agreement with the model of “tuned suppression” [63,64]. It is, therefore, conceivable that during sepsis (1) memory B cells are re-activated and that (2) tuned suppression may favor the establishment of the resulting plasma cells in the spleen. Considering the reduced anti-OVA IgG concentrations in the serum, however, it is obvious that the net effect of sepsis is an impairment of humoral memory.

In conclusion, sepsis impairs the pre-existing humoral memory in mice. We observed a decrease in antigen-specific serum antibody concentration and the number of antigen-specific bone marrow plasma cells of septic animals remained lower than in control animals. Altogether 4 wk after the induction of sepsis, there were only half as many antigen-specific bone marrow plasma cells in septic mice compared with immunized non-septic animals. The mechanism by which sepsis impedes the antigen-specific plasma cell population remains to be elucidated. Our data demonstrate, however, that in borderline cases, sepsis could endanger protective immunity.

## Supporting Information

Table S1 Influence of experimental sepsis on antibody secreting cells and serum IgG concentrations. (DOCX)
