# Supplementary material for: Experimental Sepsis Impairs Humoral Memory in Mice
Source: PLoS One. 2013 Nov 28;8(11):e81752. doi: 10.1371/journal.pone.0081752 (PMC3842948; doi:10.1371/journal.pone.0081752)
Supplement: Table S1 — (DOCX) [file pone.0081752.s001.docx]

Supporting material

**Table S1:**
Influence of experimental sepsis on antibody secreting cells and serum IgG concentrations

| OVA-specific … | Organ,  time after  CASP induction | Immunized  Median  (25%-75% percentile) | Immunized + CASP  Median  (25%-75% percentile) | *P*^a^ |
| --- | --- | --- | --- | --- |
| IgG^+^ ASC/10^6^ cells | Spleen, 2 wk | 9.5  (4.8-12.3) | 17.0  (14.2-23.3) | < 0.0001  *** |
|  | Spleen, 4 wk | 11.5  (10.3-29.8) | 17  (5.7-28.5) | 0.2480  ns |
|  | Bone marrow,  2 wk | 76.5  (54.1-76.5) | 58.0  (42.5-85.8) | 0.1328  ns |
|  | Bone marrow,  4 wk | 113.0  (58.3-160.8) | 68.6  (41.8-101.3) | 0.0368  * |
| IgG concentration (au) | Serum, 2 wk | 2614  (1467- 3396) | 1583  (719- 3031) | 0.1708  ns |
|  | Serum, 4 wk | 2342  (1191-4177) | 1084  (440-1471) | 0.0068  ** |

^a)^ Two-tailed Man-Whitney U test
